# Biomarker-based treatment selection in early-stage rectal cancer to promote organ preservation

**DOI:** 10.1002/bjs.9571

**Published:** 2014-07-23

**Authors:** K J Leong, A Beggs, J James, D G Morton, G M Matthews, S P Bach

**Affiliations:** School of Cancer Sciences, Vincent Drive, University of BirminghamBirmingham, B15 2TT, UK

## Abstract

**Background:**

Total mesorectal excision (TME) remains commonplace for T1–2 rectal cancer owing to fear of undertreating a small proportion of patients with node-positive disease. Molecular stratification may predict cancer progression. It could be used to select patients for organ-preserving surgery if specific biomarkers were validated.

**Methods:**

Gene methylation was quantified using bisulphite pyrosequencing in 133 unirradiated rectal cancer TME specimens. *KRAS* mutation and microsatellite instability status were also defined. Molecular parameters were correlated with histopathological indices of disease progression. Predictive models for nodal metastasis, lymphovascular invasion (LVI) and distant metastasis were constructed using a multilevel reverse logistic regression model.

**Results:**

Methylation of the retinoic acid receptor β gene, *RARB*, and that of the checkpoint with forkhead and ring finger gene, *CHFR*, was associated with tumour stage (*RARB:* 51·9 per cent for T1–2 *versus* 33·9 per cent for T3–4, *P* < 0·001; *CHFR:* 5·5 per cent for T1–2 *versus* 12·6 per cent for T3–4, *P* = 0·005). Gene methylation associated with nodal metastasis included *RARB* (47·1 per cent for N− *versus* 31·7 per cent for N+; *P* = 0·008), chemokine ligand 12, *CXCL12* (12·3 per cent for N− *versus* 8·9 per cent for N+; *P* = 0·021), and death-associated protein kinase 1, *DAPK1* (19·3 per cent for N− *versus* 12·3 per cent for N+; *P* = 0·022). *RARB* methylation was also associated with LVI (45·1 per cent for LVI− *versus* 31·7 per cent for LVI+; *P* = 0·038). Predictive models for nodal metastasis and LVI achieved sensitivities of 91·1 and 85·0 per cent, and specificities of 55·3 and 45·3 per cent, respectively.

**Conclusion:**

This methylation biomarker panel provides a step towards accurate discrimination of indolent and aggressive rectal cancer subtypes. This could offer an improvement over the current standard of care, whereby fit patients are offered radical surgery. May assist selection for organ preservation

## Introduction

Rectum-preserving surgery has been proposed with gradual acceptance for early-stage tumours and for those downstaged by neoadjuvant therapy^1^. Local excision, either alone or combined with radiotherapy, cures the majority of cases that appear confined to the bowel wall. However, even in this select group, recurrence rates as high as 30 per cent following organ-preserving treatment have been described^2^. Optimized organ preservation approaches would benefit from the identification of high-risk tumour characteristics other than the somewhat crude morphometric, radiological and histological stratification currently available^3^–^5^. Prediction of nodal metastasis by size or imaging characteristics is an imprecise science, particularly following neoadjuvant therapy^6^. The probability of local recurrence after transanal endoscopic microsurgery (TEMS) for early rectal cancer may be predicted based upon tumour diameter, depth of invasion and adverse histological features^7^. This model provides practical reassurance for individuals with a predicted low risk of recurrence (less than 5 per cent), where total mesorectal excision (TME) has little to offer. As risk increases (10–25 per cent), patients face a dilemma and must trade the prospect of undertreatment or overtreatment. In addition, these determinants are not applicable to preoperative samples. Hence, molecular signatures of good (or indeed poor) prognosis accessible via tumour biopsies would be useful biomarkers to stratify risk and inform organ-preserving decisions.

Early translational research^8^,^9^ has shown that patients with tumours expressing particular molecular profiles have a poor prognosis. Epigenetic differences (for instance DNA methylation) predict recurrence of early-stage rectal cancer^10^. Two patterns of abnormal DNA methylation are observed in colorectal cancer: genome-wide hypomethylation or localized hypermethylation at or near tumour suppressor gene promoters^11^. It has been suggested^12^ that the loss of function of classical tumour suppressor genes by promoter hypermethylation is more common than by mutation. Various methylation-related mechanisms that trigger genetic changes and contribute to tumorigenesis have been described^13^–^16^. Biomarkers of tumour progression show promise in distinguishing indolent and aggressive cancer in other organs^17^,^18^. Prognostic models incorporating multiple biomarkers have been described in breast^19^ and hepatocellular^20^ cancers. The present authors have shown previously^21^ that hypermethylation of two or more genes in rectal tumours can be associated with early-stage disease. The objective of this retrospective study was to refine the prognostic utility of rectal cancer biomarkers in order to aid selection of appropriate patients for organ-preserving strategies^1^,^22^.

## Methods

### Samples

Rectal tumour samples were taken from two patient groups treated with TME at the University Hospitals of Birmingham. The first group was obtained from a tissue bank of colorectal cancer specimens from patients treated between 2001 and 2004. Samples from the core of the resected tumour had been flash-frozen by the operating surgeon immediately after surgery. Histologically confirmed tumour-free samples (5 cm or more from the tumour edge) were frozen for paired analysis. The second group was obtained from histopathology archives of formalin-fixed, paraffin-embedded rectal tumour sections from patients treated between 2005 and 2010. Patients who had received neoadjuvant chemoradiotherapy, or had a family or personal history of inflammatory or malignant bowel disease were excluded. Ethical approval was obtained from the Black Country Research Ethics Committee.

### Macrodissection, DNA extraction and bisulphite treatment

Genomic DNA extraction of fresh-frozen tissues was performed using the DNeasy® Blood & Tissue Kit (QIAGEN, Crawley, UK) according to the manufacturer's instructions. Approximately 2 mm^3^ tissue was used, and purified genomic DNA was made up to a final volume of 200 µl. DNA extracts were quantified and assessed for quality using a NanoDrop 1000 Spectrophotometer (Thermo Scientific, Loughborough, UK). Bisulphite modification of 1 µg genomic DNA was performed using the EpiTect® Bisulfite Kit (QIAGEN), according to the manufacturer's instructions.

Paraffin-embedded blocks were sectioned at 5 µm thickness and mounted on glass slides. A reference slide for each sample was stained with haematoxylin and eosin. Areas containing more than 80 per cent of tumour tissue were marked to guide sampling from adjacent, non-haematoxylin and eosin-stained sections. DNA extraction and bisulphite treatment of formalin-fixed, paraffin-embedded tissues was performed using the EpiTect® Plus Bisulfite Kit (QIAGEN), according to the manufacturer's instructions.

### Bisulphite pyrosequencing

Oestrogen receptor 1 gene (*ESR1*) and unc-5 homologue C gene (*UNC5C*) assays were purchased from QIAGEN. The glutathione *S*-transferase pi 1 gene (*GSTP1*) primer sequences and polymerase chain reaction (PCR) conditions are available at the PyroMark™ assay database (http://techsupport.pyrosequencing.com). Retinoic acid receptor β (*RARB*), long interspersed nucleotide element 1 (*LINE-1*) and adenomatous polyposis coli (*APC*) gene assays have been described previously^21^,^23^,^24^. Primers for the remaining nine assays were designed using the MethPrimer software (http://www.urogene.org/methprimer/index1.html). Primer sequences and the PCR annealing temperatures (Tm), and the number of cytosine–guanine nucleotide (CpG) sites examined are shown in *Table S1* (supporting information). PCR for assays designed in-house was performed using 1 µl bisulphite-treated DNA, 5 pmol forward and reverse primers, 5 µl 10 × buffer, 1.5 µl 50-mmol/l magnesium chloride, 2·5 mmol of each dNTP and 1·5 units IMMOLASE™ DNA polymerase (Bioline, London, UK), made up to 50 µl with sterile distilled water. The PCR conditions were: 95°C for 10 min; 45 cycles of 95°C for 20 s, Tm for 20 s and 72°C for 30 s; and final extension at 72°C for 5 min. PCR for the remaining assays was performed according to either the manufacturer's instructions or published protocols (*LINE-1*). Negative water controls were included to ensure no contamination.

Some 5 µl PCR products were analysed on a 1 per cent agarose gel before pyrosequencing. Remaining PCR products were captured on streptavidin-coated beads, denatured and washed, followed by the addition of sequencing primer. Pyrosequencing was performed using PyroMark™ Gold Q96 reagents on a PyroMark™ Q96 ID machine (QIAGEN). Samples were repeated on different days to assess reproducibility. Internal validation was performed using bisulphite-treated unmethylated and methylated genomic DNA (CpGenome™ Universal DNA; Merck–Millipore, Watford, UK). The instrument software (Pyro Q-CpG™; QIAGEN) automatically calculates the percentage methylation at each CpG site in the assay by quantifying the relative peak heights of thymine/cytosine. Methylation for each gene was expressed as the median percentage methylation across all CpG sites.

### Analysis of *KRAS* mutation

*KRAS* mutational analysis at codons 12 and 13 was performed using pyrosequencing, as described previously^25^,^26^. PCR reactions of 50 µl were set up using a standard protocol of 40 cycles at Tm of 59°C using 0.3 µl MyTaq™ (Bioline) enzyme. PCR products were processed and run on the PyroMark™ Q96 ID machine. The software automatically generates percentage values for codon 12 and 13 single-nucleotide polymorphisms. Nucleotide substitutions greater than 2·5 per cent were considered indicative of *KRAS* mutation.

### Analysis of microsatellite instability status

Microsatellite instability (MSI) status was analysed using a refined method of the ‘Bethesda panel’ of markers^27^. The modified panel consists of mononucleotide repeat markers NR-21, NR-24, NR-27, BAT-25 and BAT-26. Tumours with instability at two or more markers were defined as MSI-high, whereas tumours with single-marker instability were designated as MSI-low.

Relevant marker sections were amplified in a 25-µl multiplex PCR reaction including 1 µl sample DNA (35 cycles, Tm 55°C, 0·2 µl MyTaq™ enzyme). Fragment analysis was performed on an ABI 3730 machine (Life Technologies, Paisley, UK). Microsatellite marker sizes were determined using Peak Scanner™ software (Life Technologies).

### Statistical analysis

Statistical significance between groups was determined using a Wilcoxon signed rank test for paired variables, Mann–Whitney *U* test for unpaired continuous variables, and Fisher's exact test for categorical variables. *P* < 0·050 was considered statistically significant.

To design a predictive model using percentage methylation of individual CpGs for each gene of interest, a two-step logistic regression model was undertaken using Stata® version 12.1 (StataCorp LP, College Station, Texas, USA). This was done to predict lymph node status, lymphovascular invasion (LVI) and distant metastasis – histopathological parameters considered to be associated with adverse patient outcomes. In the first step, a reverse stepwise logistic regression model was performed using CpGs in each gene as independent variables. The threshold for significance was set at *P* < 0·100; significant CpGs were taken forward into the next stage, where these CpGs were entered into a reverse stepwise logistic regression model as independent variables, with the significance threshold for removal from the model set at *P* < 0·100. MSI status and *KRAS* mutation status were also entered into the model. The fit of the model was modelled using the area under the curve (AUC) statistic and a model was considered a good predictor when the AUC was above 0·70. Goodness-of-fit testing was carried out using standard residuals plots and the Hosmer–Lemeshow test (with *P* < 0·100 indicating an inadequate model). Model threshold for prediction was set pragmatically, aiming to maximize sensitivity as close as possible to 90 per cent or above, and keeping specificity close to 50 per cent or above, on the basis that high-frequency detection of adverse features was more important than inclusion of patients without these features within the cohort.

## Results

**Fig 1:**
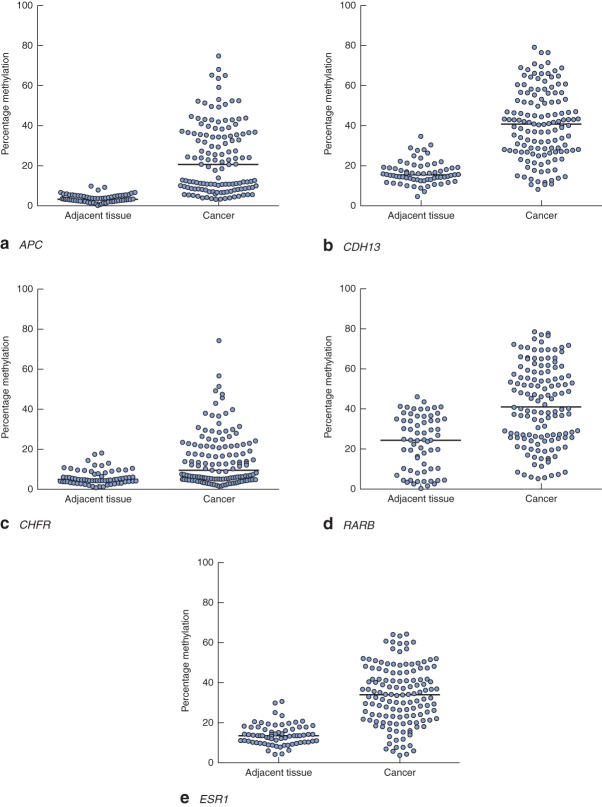
Methylation levels of **a** *APC*, **b** *CDH13*, **c** *CHFR*, **d** *RARB* and **e** *ESR1* genes in matched adjacent tissues and rectal cancers. Horizontal bars represent median methylation levels. **a–e** *P* < 0·001 (2-tailed Wilcoxon signed rank test)

**Fig 2:**
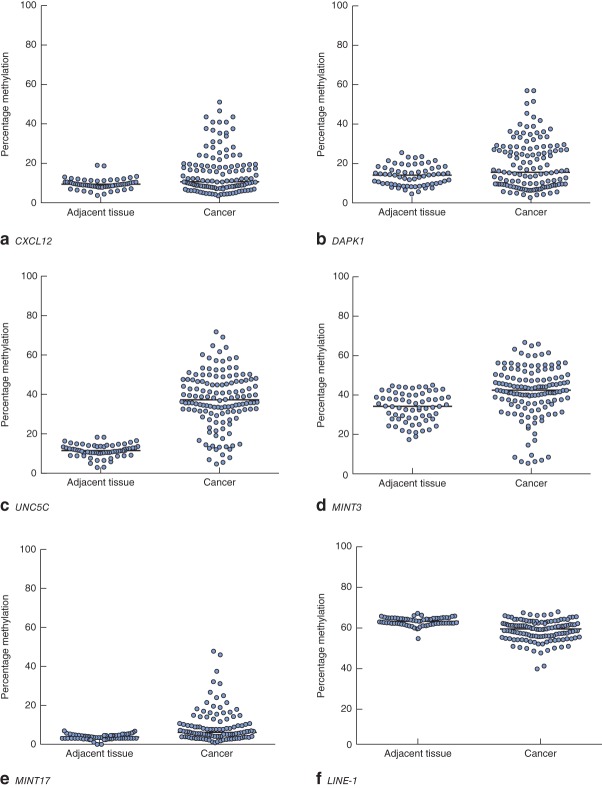
Methylation levels of **a** *CXCL12*, **b** *DAPK1*, **c** *UNC5C*, **d** *MINT3*, **e** *MINT17* and **f** *LINE-1* genes in matched adjacent tissues and rectal cancers. Horizontal bars represent median methylation levels. **a,c–f** *P* < 0·001, **b** *P* = 0·007 (2-tailed Wilcoxon signed rank test)

**Fig 3:**
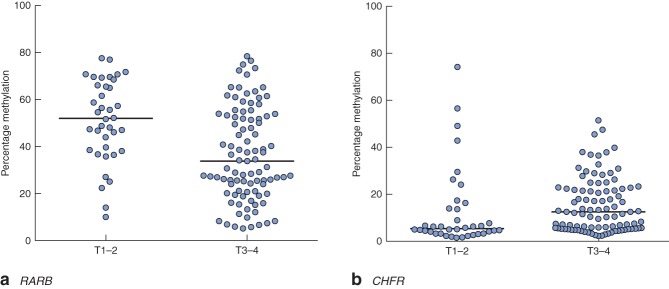
Methylation levels of **a** *RARB* and **b** *CHFR* genes, stratified according to depth of invasion. Horizontal bars represent median methylation levels. **a** *P* < 0·001, **b** *P* = 0·005 (2-tailed Mann–Whitney *U* test)

**Fig 4:**
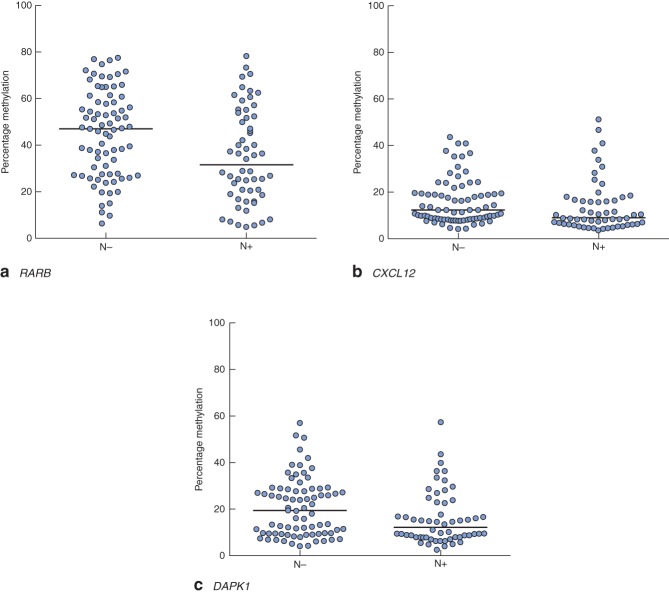
Methylation levels of **a** *RARB*, **b** *CXCL12* and **c** *DAPK1* genes, stratified according to nodal metastasis: N−, nodal metastasis absent; N+, nodal metastasis present. Horizontal bars represent median methylation levels. **a** *P* = 0·008, **b** *P* = 0·021, **c** *P* = 0·022 (2-tailed Mann–Whitney *U* test)

**Fig 5:**
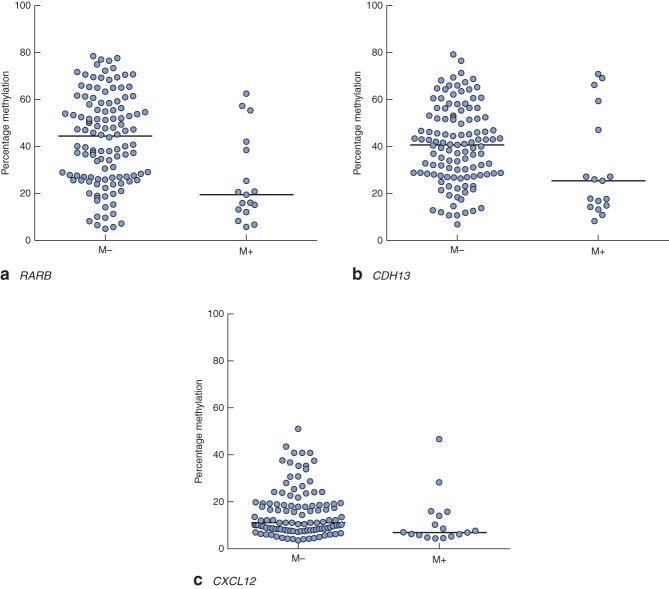
Methylation levels of **a** *RARB*, **b** *CDH13* and **c** *CXCL12* genes, stratified according to distant metastasis: M−, distant metastasis absent; M+, distant metastasis present. Horizontal bars represent median methylation levels. **a** *P* < 0·001, **b** *P* = 0·027, **c** *P* = 0·018 (2-tailed Mann–Whitney *U* test)

**Fig 6:**
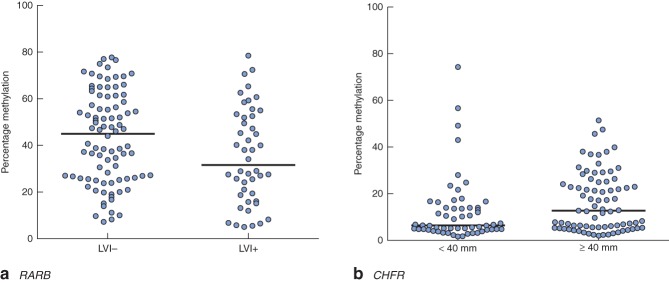
Methylation levels of **a** *RARB* and **b** *CHFR* genes, stratified according to **a** lymphovascular invasion (LVI; absent or present) and **b** tumour size (less than, or greater than or equal to the median tumour size of 40 mm). Horizontal bars represent median methylation levels. **a** *P* = 0·038, **b** *P* = 0·011 (2-tailed Mann–Whitney *U* test)

Clinical and pathological data are summarized in Table  1. Gene-specific hypermethylation was observed in rectal cancer, compared with matched adjacent normal mucosa (*Figs* 1 and 2; *Fig. S1*, supporting information). As expected, the methylation level of *LINE-1*, a marker of global methylation, was lower in rectal cancer (*Fig.* 2). Median percentage methylation was calculated for each of 15 genes for analysis of association with tumour stage and pathology variables (*Figs* 6). Early-stage (pathological tumour (pT) 1–2) and node-negative tumours had higher median percentage methylation for *RARB* (*Figs* 3 and 4). Larger tumours (median diameter 40 mm or more) had higher methylation levels of checkpoint with forkhead and ring finger gene, *CHFR* (*Fig.* 6), as did more advanced lesions (pT3–4). Node-negative tumours also exhibited increased methylation levels of chemokine ligand 12 gene (*CXCL12*) and death-associated protein kinase 1 gene (*DAPK1*) compared with node-positive tumours (*Fig.* 4). Tumours with either LVI or distant metastasis were associated with lower methylation values of *RARB* (*Figs* 5 and 6). In addition, tumours with organ secondaries were associated with less methylation of cadherin 13 gene, *CDH13*, and *CXCL12* (*Fig.* 5).

**Table 1:** Patient demographics and clinicopathological characteristics of samples used

	Adjacent tissue(*n* = 64)	Rectal cancer(*n* = 133)
Age (years)*	71 (33–89)	70 (31–92)
Sex ratio (M : F)	42 : 22	78 : 55
TNM stage		
I		32 (24·1)
II		43 (32·3)
III		41 (30·8)
IV		17 (12·8)
Tumour size (mm)*		40 (9–110)
Vascular invasion		
No		87 (65·4)
Yes		46 (34·6)
Degree of differentiation		
Well		12 (9·0)
Moderate		115 (86·5)
Poor		6 (4·5)

Values in parentheses are percentages unless indicated otherwise

* values are median (range). TNM, tumour node metastasis classification.

One-third of rectal cancers (44 of 133) carried the *KRAS* mutation at codon 12 or 13. In contrast, only six rectal tumours were found to be microsatellite unstable (3 MSI-high and 3 MSI-low). Neither *KRAS* mutation nor MSI status was associated with advanced disease in univariable analysis.

### Construction of a multivariable biomarker model to predict disease progression in rectal cancer

Bisulphite pyrosequencing can quantify methylation at multiple sequential CpGs, a feature that is absent in other methylation platforms. Interrogation of individual CpGs, rather than the mean across a locus, may identify the most important or useful sites. A multilevel reverse stepwise logistic regression model was constructed using individual CpGs to identify genes associated with disease progression: LVI, lymph node metastasis and distant metastasis (*Table S2*, supporting information).

**Fig 7:**
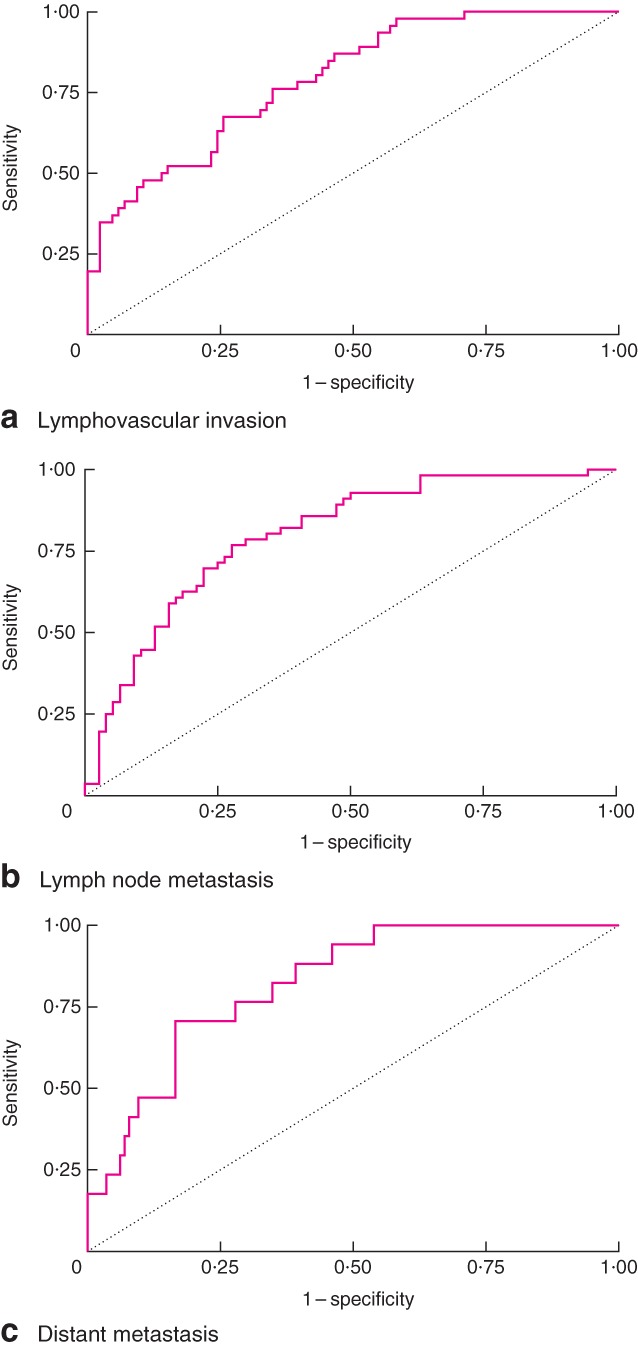
Receiver operating characteristic (ROC) curves constructed for **a** lymphovascular invasion, **b** lymph node metastasis and **c** distant metastasis. Area under ROC curve: **a** 0·76, **b** 0·76, **c** 0·82

For LVI, the model identified seven CpGs from three genes as the most informative variables: *CDH1* (sites 1, 2 and 4), *CDH13* (sites 2 and 5) and methylated-in-tumour 3, *MINT3* (sites 6 and 9) (Table 2). A model score was developed by summating the percentage methylation of each CpG, maintaining the direction of the coefficient. The AUC for this model was 0·76 (95 per cent confidence interval (c.i.) 0·68 to 0·84), and setting a cut-off score of −20 gave a sensitivity of 85·0 per cent, specificity of 45·3 per cent with a positive predictive value of (PPV) of 62·5 per cent and a negative predictive value (NPV) of 74·0 per cent (*Fig.* 7).

**Table 2:** Biomarker model of genes associated with histopathological features of disease progression

Histopathological feature	Genes
Lymphovascular invasion	*CDH1*, *CDH13*, *MINT3*
Lymph node metastasis	*CDH1*, *CDH13*, *MINT3*, *CXCL12*, *RARB*, *APC*
Distant metastasis	*CDH1*, *MINT3*, *CXCL12*, *RARB*, *ESR1*, *CHFR*

For lymph node metastasis, ten CpGs from six genes were found to be significant: *CDH1* (site 10)*, CDH13* (sites 7 and 8)*, MINT3* (sites 3 and 4), *CXCL12* (sites 1 and 3)*, RARB* (site 6) and *APC* (sites 1 and 6) (Table 2)*.* The AUC for the model was found to be 0·76 (95 per cent c.i. 0·68 to 0·84), and setting a cut-off score of −43 gave a sensitivity of 91·1 per cent, specificity of 55·3 per cent, PPV of 60·0 per cent and NPV of 89·4 per cent (*Fig.* 7).

For distant metastasis, nine CpGs from six genes were selected from the model: *CDH1* (sites 2 and 6)*, MINT3* (sites 6 and 8), *CXCL12* (sites 1 and 3), *RARB* (site 5), *ESR1* (site 4) and *CHFR* (site 6) (*Table* 2). The AUC was 0·82 (95 per cent c.i. 0·73 to 0·91), and use of a cut-off score of −40 gave a sensitivity of 100 per cent and specificity of 51·3 per cent, with a PPV of 100 per cent and a NPV of 90·6 per cent (*Fig.* 7).

## Discussion

Clinical outcomes for early rectal cancer are heterogeneous and it is becoming clear that genetic events play a role in determining such variation. Following on from previous work^21^ showing an association between gene methylation and pathological indices of disease progression, the present study explored its feasibility as a predictive biomarker. The results suggest that a multimarker methylation model is needed to achieve high sensitivity for the prediction of disease progression. Single-gene testing is unlikely to be clinically useful for prognosis; although MSI immunohistochemistry is becoming routine and may confer prognostic significance in certain scenarios^28^, it is notable that multiple mutations are tested simultaneously.

The majority of early rectal cancers are node-negative, and routine removal of the mesorectum may be unnecessary. Deciding which patient is suitable for an organ-preserving approach requires more refined prognostic testing than is currently available. A pragmatic approach was undertaken when determining the sensitivity and specificity thresholds for the present predictive model. The aim was to achieve a high sensitivity (90 per cent or above) for patients at risk of disease progression with a reasonable specificity (close to 50 per cent). By stratifying 50 per cent of patients as high risk and 50 per cent as low risk, organ-preserving treatments could be offered to the latter according to the profile of a small subset of genes. Analysis of the methylome using next-generation sequencing or gene array technologies may reveal additional markers to stratify risk further and tailor treatment options (radiotherapy, local excision or radical surgery).

The present work has provided a step towards accurate discrimination of indolent and aggressive rectal cancer subtypes. The next stage is to evaluate the present biomarker model in a prospective study. It can be tested as part of the Transanal Endoscopic Microsurgery and Radiotherapy in Early Rectal Cancer (TREC) study, which is already evaluating the feasibility of randomization between conventional surgery and a new organ-preserving protocol of short-course preoperative radiotherapy with a 10-week interval to TEMS^29^. Correlation of the molecular analysis with clinical outcome data will be required to determine the predictive accuracy of the present biomarker model.

## Supporting information

Table S1 Primer sequences, PCR annealing temperatures of pyrosequencing assays and number of CpG sites examined (Word document)

Table S2 Significant genes and their corresponding CpG sites for the prediction of lymph node involvement, lymphovascular invasion and distant metastasis in rectal cancer (Word document)

Fig S1 Methylation levels of *CDH1*, *GSTP1*, *CASP8* and *TIMP3* genes in adjacent tissues and rectal cancers (TIFF file)
