# Supplementary material for: Biomarker-based treatment selection in early-stage rectal cancer to promote organ preservation
Source: Br J Surg. 2014 Jul 23;101(10):1299–309. doi: 10.1002/bjs.9571 (PMC4282074; doi:10.1002/bjs.9571)
Supplement: Table S1 — Primer sequences, PCR annealing temperatures of pyrosequencing assays and number of CpG sites examined (Word document) [file bjs0101-1299-SD1.doc]

**Table S1** Primer sequences, PCR annealing temperatures of pyrosequencing assays and number of CpG sites examined

| Gene | Primers | | Tm (°C) | No. of CpG sites |
| --- | --- | --- | --- | --- |
| *CDH13* | Forward | TTGGAAAAGTGGAATTAGTTGGTAT | 56 | 9 |
|  | Reverse | (B)ACCAAAACCAATAACTTTACAAAAC |  |  |
|  | Sequencing | AAAGAAGTAAATGGGATGTTATTTT |  |  |
|  |  |  |  |  |
| *CHFR* | Forward | TAGAATTTTTGGGGTTTTTAATT | 56 | 10 |
|  | Reverse | (B)ACCATCTTTAATCCTAACCAAAC |  |  |
|  | Sequencing | TAGAATTTTTGGGGTTTTTAATT |  |  |
|  |  |  |  |  |
| *CXCL12* | Forward | GGGATTAATTTGTTTGTTTTTTATTG | 58 | 7 |
|  | Reverse | (B)ACCTTTAACCTTCTCAAACTCC |  |  |
|  | Sequencing | TGTTTTTTATTGGTTTTTATTTAGTTT |  |  |
|  |  |  |  |  |
| *DAPK1* | Forward | TTTTGGAGGTGGGAAAGTTG | 55 | 10 |
|  | Reverse | (B)AAAAACACCCTTTATTAAAACTAAAC |  |  |
|  | Sequencing | GGGTATGTGTGTAGAGAAAGGGGA |  |  |
|  |  |  |  |  |
| *MINT3* | Forward | TGATGGTGTATGTGATTTTGTGTT | 60 | 10 |
|  | Reverse | (B)ACCCCACCCCTCACAAAC |  |  |
|  | Sequencing | TGATGGTGTATGTGATTTTGTGTT |  |  |
|  |  |  |  |  |
| *MINT17* | Forward | AGGGGTTAGGTTGAGGTTGTT | 58 | 4 |
|  | Reverse | (B)TCTACCTCTTCCCAAATTCCA |  |  |
|  | Sequencing | AGGGGTTAGGTTGAGGTTGTT |  |  |
|  |  |  |  |  |
| *CDH1* | Forward | TTTTAGTAATTTTAGGTTAGAGGGTTAT | 56 | 10 |
|  | Reverse | (B)TAACTACAACCAAATAAACCCC |  |  |
|  | Sequencing | TTTTAGTAATTTTAGGTTAGAGGGTTAT |  |  |
|  |  |  |  |  |
| CASP8 | Forward | TTAATAGGAAGTGAGGTTATGGAGG | 59 | 4 |
|  | Reverse | (B)TAAACCAAACAACACCCAAAAATAT |  |  |
|  | Sequencing | ATTTTTTTTTTTGTTGAGTA |  |  |
|  |  |  |  |  |
| *TIMP3* | Forward | TGGTTTGGGTTAGAGATATTTAGTG | 59 | 5 |
|  | Reverse | (B)CCCCCTCAAACCAATAACAA |  |  |
|  | Sequencing | ATTTTTTATAAGGATTTGAA |  |  |

PCR, polymerase chain reaction; Tm, annealing temperature; (B), biotinylated.
