# Supplementary material for: Biomarker-based treatment selection in early-stage rectal cancer to promote organ preservation
Source: Br J Surg. 2014 Jul 23;101(10):1299–309. doi: 10.1002/bjs.9571 (PMC4282074; doi:10.1002/bjs.9571)
Supplement: Table S2 — Significant genes and their corresponding CpG sites for the prediction of lymph node involvement, lymphovascular invasion and distant metastasis in rectal cancer (Word document) [file bjs0101-1299-SD2.doc]

**Table S2** Significant genes and their corresponding CpG sites for the prediction of lymph node involvement, lymphovascular invasion and distant metastasis in rectal cancer

| Gene | CpG | Coefficient | 95% c.i. | *z* | *P* > *z* |
| --- | --- | --- | --- | --- | --- |
| Lymph node involvement |  |  |  |  |  |
| *APC* | 1 | 0.11 | 0.01, 0.20 | 2.27 | 0.023 |
|  | 6 | −0.10 | −0.17, −0.03 | −2.31 | 0.021 |
| *CDH13* | 7 | −0.09 | −0.15, −0.03 | −2.44 | 0.015 |
|  | 8 | 0.09 | 0.02, 0.16 | 2.46 | 0.014 |
| *CXCL12* | 1 | 0.11 | 0.04, 0.19 | 3.03 | 0.002 |
|  | 3 | −0.11 | −0.16, −0.06 | −2.79 | 0.005 |
| *CDH1* | 10 | 0.07 | 0, 0.14 | 1.99 | 0.047 |
| *MINT3* | 3 | −0.18 | −0.33, −0.03 | −2.1 | 0.035 |
|  | 4 | 0.19 | 0.02, 0.37 | 2.19 | 0.029 |
| *RARB* | 6 | −0.03 | −0.05, −0.01 | −2.42 | 0.016 |
| Lymphovascular invasion |  |  |  |  |  |
| *CDH1* | 1 | 0.27 | 0.06, 0.49 | 2.47 | 0.014 |
|  | 2 | −0.25 | −0.51, −0.01 | −1.9 | 0.048 |
|  | 4 | −0.14 | −0.22, −0.02 | −2.48 | 0.013 |
| *CDH13* | 2 | 0.14 | 0.05, 0.23 | 3.03 | 0.002 |
|  | 5 | −0.18 | −0.22, −0.14 | −3.22 | 0.001 |
| *MINT3* | 6 | −0.38 | −0.40, −0.36 | −3.67 | < 0.001 |
|  | 9 | 0.45 | 0.21, 0.70 | 3.6 | < 0.001 |
| Distant metastasis |  |  |  |  |  |
| *CHFR* | 6 | −0.09 | −0.13, −0.05 | −2.66 | 0.008 |
| *CXCL12* | 1 | 0.12 | 0.02, 0.22 | 2.34 | 0.019 |
|  | 3 | −0.11 | −0.22, −0.01 | −2.04 | 0.041 |
| *CDH1* | 2 | −0.57 | −0.83, −0.31 | −2.66 | 0.008 |
|  | 6 | 0.37 | 0.12, 0.63 | 2.86 | 0.004 |
| *ESR1* | 4 | 0.07 | 0.01, 0.13 | 2.37 | 0.018 |
| *MINT3* | 6 | −0.21 | −0.50, −0.01 | −1.68 | 0.039 |
|  | 8 | 0.22 | −0.51, 0.93 | 1.75 | 0.049 |
| *RARB* | 5 | −0.06 | −0.08, −0.04 | −3.06 | 0.002 |

c.i., confidence interval.
