# Supplementary figures and images for: Biomarker-based treatment selection in early-stage rectal cancer to promote organ preservation
Source: Br J Surg. 2014 Jul 23;101(10):1299–309. doi: 10.1002/bjs.9571 (PMC4282074; doi:10.1002/bjs.9571)

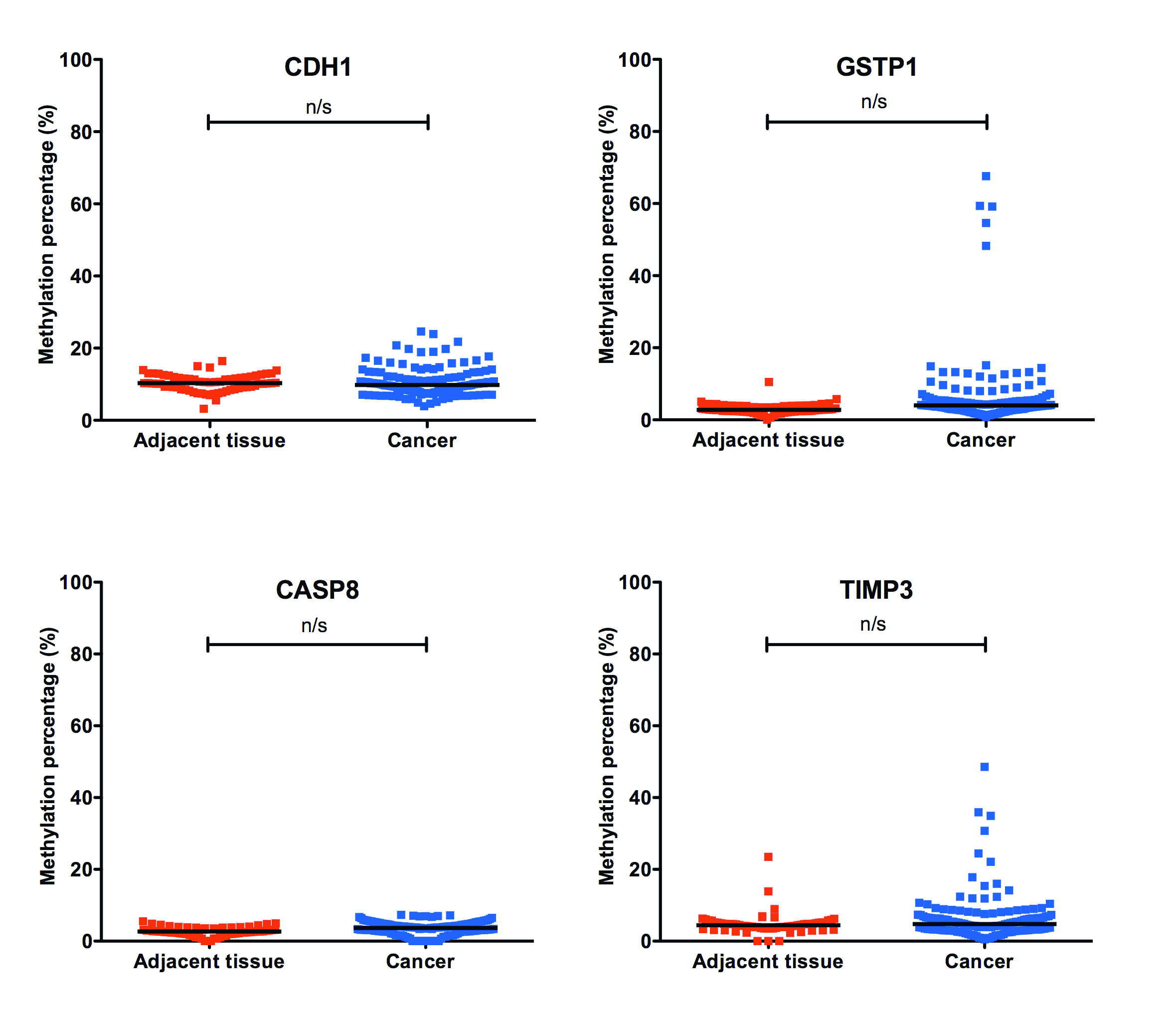

Supplement: Fig S1 — Methylation levels of CDH1, GSTP1, CASP8 and TIMP3 genes in adjacent tissues and rectal cancers (TIFF file) [file bjs0101-1299-SD3.tiff]
